# Remitting Seronegative Symmetrical Synovitis With Pitting Oedema Associated With Malignancy: A Case Report

**DOI:** 10.7759/cureus.62980

**Published:** 2024-06-23

**Authors:** Maria Inês Matos, João Rocha, Mariana Matos, Maria Teresa Brito, Susana Ferreira

**Affiliations:** 1 Internal Medicine, Centro Hospitalar Universitário de São João, Porto, PRT

**Keywords:** seronegative inflammatory polyarthritis, polymyalgia rheumatica, clear renal cell carcinoma, elderly patients, remitting seronegative symmetrical synovitis with pitting edema

## Abstract

Remitting seronegative symmetrical synovitis with pitting oedema is a rare rheumatological condition, predominating in the elderly male. It is characterised by the abrupt onset of marked pitting oedema, symmetrical distal synovitis, absence of rheumatoid factor and an excellent response to glucocorticoids. RS3PE may be the harbinger of a malignancy so the diagnosis should prompt evaluation and exclusion of such condition; in these cases, the response to glucocorticoids is only partial and treating the neoplasia is essential. The differential diagnosis includes late-onset rheumatoid arthritis, polymyalgia rheumatica and calcium pyrophosphate crystal-related arthritis. We present the case of a patient with remitting seronegative symmetrical synovitis with pitting oedema associated with clear cell renal cell carcinoma.

## Introduction

Remitting seronegative symmetrical synovitis with pitting oedema (RS3PE) is a rare rheumatological condition [1], belonging to the group of seronegative inflammatory polyarthritis. The pathophysiology of the disease is not completely understood. It is characterised by abrupt onset of marked pitting oedema (predominantly on the dorsal part of the hands), symmetrical distal synovitis, predominating in the elderly male, absence of rheumatoid factor and an excellent response to glucocorticoids. There is a significant association with underlying malignancy, so the diagnosis of RS3PE should prompt evaluation for such a condition [2]. This article was previously presented as a meeting abstract at the 26º Congresso Nacional de Medicina Interna, 2020.

## Case presentation

**Figure 1:**
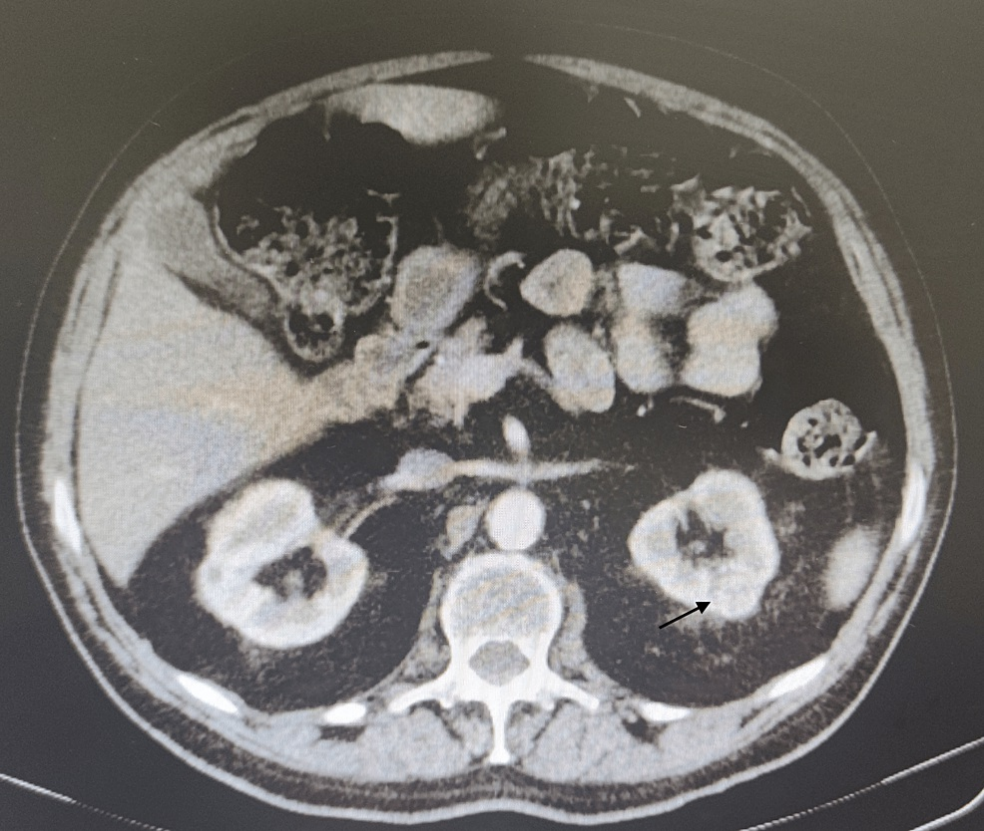
Left kidney nodule The image shows an exophytic, contrast-enhancing nodule, with 18 mm, in the left kidney, suggestive of malignancy (black arrow).

A 72-year-old male was admitted to our ward with unintentional weight loss (9% of body weight), asthenia, symmetrical swelling of wrists and ankles and bilateral pain in knees and shoulders, lasting for a month and with significant functional impairment. He was a former smoker, diagnosed with essential arterial hypertension, dyslipidaemia and type 2 diabetes mellitus, with cerebrovascular disease (posterior circulation ischaemic stroke in 2017, without sequelae). He was also diagnosed with high-grade papillary urothelial carcinoma in 2017 (stage pT1), treated with transurethral resection and intravesical Bacillus Calmette-Guerin. He had been regularly followed up in Urology consultation, without evidence of recurrence. One month before admission, a routine CT scan of the abdomen and pelvis (Figure 1) was electively performed, with identification of an exophytic, enhancing nodule in the left kidney (18 mm in diameter), waiting for further evaluation by Urology.

On the physical examination, the evaluation of pulmonary, cardiovascular, genitourinary and gastrointestinal systems was unremarkable. Significant pitting oedema was found on the dorsal part of the hands. Periods of fever were documented. Neurologic examination showed bilateral decreased proximal muscular strength in the upper limbs, with painful and limited shoulder, wrist and ankle movements. Temporal pulses were normal and not tender to palpation. There was no Raynaud’s phenomenon or skin lesions.

Laboratory workup revealed normocytic normochromic anaemia (haemoglobin 11.7 g/dL), white blood cell count 7,100/μL, platelet count 170,000/μL, erythrocyte sedimentation rate 62 mm/h and C-reactive protein 33.2 mg/L. Liver, kidney and thyroid function tests showed no abnormalities. No increase of creatine kinase or myoglobin was found. No significant alterations were registered in serum protein electrophoresis and immunoglobulins’ levels. Rheumatoid factor (RF), antinuclear, anti-double-stranded DNA and anti-citrullinated protein antibodies (ACPA) were negative. No microbiological agents were found in peripheral blood and urine cultures, and HIV, HCV and HBV antibodies were negative.

Hand x-ray showed absence of joints erosions. Thoracoabdominal CT scan revealed an exophytic, contrast-enhanced nodule in the left kidney, already present in the prior study. No other proliferative lesions or adenopathy was found. Urine cytology was negative for malignant cells. Cystoscopy showed no vesical lesions. Upper endoscopy showed no significant lesions, and colonoscopy revealed a rectal sessile polyp, later identified as a tubulovillous adenoma with low-grade dysplasia lesions on pathological examination. No signs of polyneuropathy, myopathy or neurodegenerative disease were found on electromyography. Bone scintigraphy revealed increased uptake in dorso-lumbar, sternoclavicular, shoulder, elbow, carpal, metacarpophalangeal and knee joints, suggesting degenerative lesions, and no signs of bone metastasis were found.

The diagnosis of RS3PE was made, and paraneoplastic syndrome related to kidney neoplasm was suggested. The patient was given 10 mg per day of prednisolone, with significant improvement in wrists, constitutional symptoms, pain and joint mobility, as well as decreased levels of inflammatory markers. He was discharged maintaining the same prednisolone dosage, considering the persistence of neoplastic kidney lesion (not treated at the time) as well as persistence of symptoms.

The patient was followed up in Internal Medicine consultation, without recurrence of symptoms and signs related to RS3PE with the prescribed dosage of prednisolone. After Urology evaluation, a partial left nephrectomy was performed, and clear cell renal carcinoma was diagnosed on pathological examination (PT1a N0 M0 R0, grade 2). Slow prednisolone tapering, until full suspension, was performed, and the patient remained free of relapsing symptoms and signs of RS3PE.

## Discussion

RS3PE was first described in 1985 by McCarty et al. [1], who used the term “boxing glove hand” due to the marked pitting oedema of the hands associated with the condition. He characterised the condition as a sudden onset of polyarthritis associated with pitting oedema on the dorsal part of both hands and/or feet, with negative rheumatoid factor (RF), and the absence of erosions on radiography. The polyarthritis is more commonly bilateral, symmetrical, and with a preference for the distal limbs, primarily affecting the joints of the wrist, metacarpophalangeal, proximal interphalangeal and ankle. Several studies have demonstrated a male preponderance (2:1). The condition is more frequent in the elderly, and constitutional symptoms are common [2]. Most cases are associated with tenosynovitis of the extensor tendons of the hands [3]. In 2016, the authors of a systematic review and meta-analysis proposed the following diagnostic criteria [2]: abrupt onset; marked pitting oedema of mostly hands (and/or feet); age of onset ≥ 60 years; good response to a short course of medium-dose steroids (10-20 mg); seronegative for RF and ACPA; and absence of radiographic joint erosions.

Laboratory findings include various degrees of anaemia of chronic disease, elevated acute-phase reactants (such as erythrocyte sedimentation rate and serum C-reactive protein) and negative RF and ACPA. Increased levels of vascular endothelial growth factor have been reported; however, a recent multicentre study [4] found that this increase occurs in most rheumatic diseases and is not associated with this specific condition. Imaging modalities such as ultrasound and magnetic resonance imaging allow the visualisation of subcutaneous oedema and extensor tenosynovitis which represent the hallmark of the condition.

Many studies have reported an association of RS3PE with solid (prostate, gastric, intestine, lung, ovarian, bladder, breast and endometrial) and haematological malignancies (leukaemia, myelodysplastic syndrome and lymphoma), with the prevalence being between 16% and 30% [2,5]. RS3PE may be the harbinger of a malignancy so the diagnosis should prompt evaluation, starting with age-appropriate cancer screening and a careful and complete history focusing on symptoms that may suggest an underlying malignancy.

Some authors have considered RS3PE to be a subset of rheumatoid arthritis; however, the lack of positivity for RF and ACPA, the absence of erosions, the male and elderly preponderance and the excellent prognosis with low doses of glucocorticoids have made it clear that this is a distinct entity [6]. Similarly, some authors considered the condition has a forme fruste of polymyalgia rheumatica, but there are many distinctive features including male preponderance, predominant involvement of distal extremities, shorter duration of symptoms , presence of pitting oedema and very low recurrence rates. The differential diagnosis includes late-onset rheumatoid arthritis, seronegative elderly-onset rheumatoid arthritis, polymyalgia rheumatica and calcium pyrophosphate crystal-related arthritis.

Treatment consists of low-dose glucocorticoids, with most patients having an excellent and rapid response, with very low recurrence rates [7]. Patients with concurrent malignancy are more likely to have diminished and/or delayed responses to therapy, with higher rates of relapse; although glucocorticoids are still important in these patients, treatment of the underlying malignancy is associated with resolution of symptoms [2,8]. To the knowledge of the authors, this is the second case of RS3PE associated with clear cell renal cell carcinoma described in the literature [9].

## Conclusions

Our case fulfilled all of the premises mentioned: the patient was an elderly male, who presented with marked pitting oedema of the hands, had increased acute-phase reactants and seronegativity for RF and ACPA and improved only partially with corticotherapy. During the investigation, a clear cell renal cell carcinoma was diagnosed, with complete remission of symptoms being achieved with surgical removal of the kidney mass, with no need to maintain chronic corticotherapy. This case highlights the importance of excluding and treating malignancy in these patients and the value of the diagnostic criteria.
